# Milk NIR spectroscopy and Aquaphotomics novel diagnostic approach to Paratuberculosis in dairy cattle

**DOI:** 10.1038/s41598-025-99421-x

**Published:** 2025-06-02

**Authors:** Saba Behdad, Reza Massudi, Abbas Pakdel, Sahereh Joezy Shekalgorabi

**Affiliations:** 1 Department of Animal Science, College of Agriculture, Science and Research Branch, Islamic Azad University, Tehran, 14778-93855 Iran; 2 Laser and Plasma Research Institute, Shahid Beheshti University, Tehran, 19839-69411 Iran; 3 Department of Animal Science, College of Agriculture, Isfahan University of Technology, Isfahan, 84156- 83111 Iran; 4 Department of Animal Science, College of Agriculture, Shahr-e-Qods Branch, Islamic Azad University, Tehran, Iran

**Keywords:** Biophysics, Chemical biology, Developmental biology, Immunology

## Abstract

*Mycobacterium Avium* subspecies *Paratuberculosis* (MAP) causes Johne’s disease or Paratuberculosis, a chronic, progressive intestinal disease in ruminants. The incidence and prevalence of Johne’s disease are higher in dairy cattle herds because of intensive breeding and high production. Developing non-destructive diagnostic methods for early detection of this disease by simple sampling is paramount for breeding, economic, and health programs. Conventional methods are almost entirely destructive, have low accuracy, and are time-consuming. Near- infrared spectroscopy (NIRS) and Aquaphotomics can detect changes in biofluids and thus have the potential to diagnose the disease. This study aimed to investigate the diagnostic ability of NIRS and Aquaphotomics for Paratuberculosis in dairy cattle by milk sample. Milk samples from dairy cattle were collected in the NIR range (1300–1600 nm) 60 days before and 100–200 days after calving in two groups, positive and negative, using the three same consecutive ELISA test results of blood plasma and milk, as a reference test. The NIRS and Aquaphotomics methods in quadratic discriminant analysis (QDA) and support vector machine (SVM) models achieved high accuracy in detecting negative and positive groups. In internal validation, SVM and QDA models in 12 water absorbance bands had 100% accuracy. In external validation, milk samples with blood plasma ELISA reference test achieved 100% sensitivity, which is more accurate than milk ELISA as a reference test. The current study found that monitoring milk with NIR spectra provides an opportunity to analyze antibody levels indirectly via changes in water spectral patterns caused by complex physiological changes, such as the amount of antibodies related to Paratuberculosis by aquagram.

## Introduction

*Mycobacterium avium* subspecies *Paratuberculosis* (MAP), a member of the *Mycobacterium avium complex*, causes Paratuberculosis or Johne’s disease (JD), a contagious, chronic, and usually fatal infection. Johne’s disease is classified as a group B disease, indicating that it is transmissible and is significant from socioeconomic and public health perspectives^1^. It frequently affects ruminants and can spread to the entire herd, leading to outbreaks^2,3^. One notable attribute of this strain is its ability to gain access to a susceptible host, evade host defense mechanisms, and rapidly propagate to other hosts^4^. This disease can reduce productive traits such as milk and meat production and fertility rate, significantly impacting the herd’s profitability^5^. Ruminants are infected with a weakly Gram-positive acid-fast bacterium that can spread to the entire herd via horizontal transmission, mainly through the fecal-oral route, and vertical transmission via MAP from the infected dam to the embryo by the placenta^6^.

Johne’s disease has four stages: silent, subclinical, clinical, and advanced. It may affect calves from the embryonic period to the first months of birth, although clinical symptoms may not be revealed for years. Other animals can be exposed to contamination from the feces of infected animals, the environment, food, and milk, increasing the spread of the disease in the herd, so that for each animal in the final stage of the disease, there are 1 to 2 animals in the clinical stage, 6 to 8 animals in the subclinical stage, and 15 to 25 animals in the initial stage of the disease^7^. This point emphasizes the need to pay special attention to Johne’s disease in preserving genetic resources and economic and health management, so it can be called the “Trojan horse” or simply a “Trojan”^8^.

The prevalence and incidence of JD in dairy herds are higher than beef herds due to the intensive breeding of dairy cattle. Moreover, the in beef cattle are mostly slaughtered before they become symptomatic through contracting this disease^9^. The losses of Johne’s disease contain direct and indirect effects^10^. Direct effects include visible and invisible effects. One of the visible effects of Paratuberculosis infection is lower meat and milk production^11^. In a recent meta-analysis study in infected dairy farms in Switzerland, with a Paratuberculosis prevalence of 6% in cattle of this country, a total loss of 11,095,652 € per year was calculated for a population of 559,900 dairy cows. Accordingly, milk yield reduction based on a lactation period of 305 days results in an economic loss of 4,304,577 € annually^12^. In another study, it was estimated that approximately 1% of gross milk revenue, equivalent to US$33 per cow, is lost annually in MAP-infected dairy herds. In this regard, one should also consider human infections resulting from this infection, directly or indirectly, by consumption of contaminated milk and meat^13^. This underscores the significance of understanding this infection and its impact on society’s economy and decision-making especially in milk production.

The findings indicate that different quantitative trait loci (QTLs) have been identified based on the method of disease diagnosis^14^. Based on these findings, the method of disease diagnosis greatly impacts the identification of effective QTLs in Johne’s disease and genomics studies aimed at identifying QTLs for disease resistance or susceptibility. Identification of accurate disease diagnosis methods is essential for selecting animals with the JD resistant genes.

It’s important to note that the agent responsible for Johne’s disease can be found in the meat, milk, and manure of infected animals. This agent may not be destroyed during pasteurization and may cause Crohn’s disease in humans due to the lack of early and accurate diagnostic tests, MAP’s resistance to antibiotics and disinfectants, and the need for better disease control measures. As a result, Johne’s disease has become a global challenge^15,16^.

The recent study about the effect of MAP and inflammatory bowel disease (IBD) (including Crohn’s disease and Ulcerative Colitis) introduced a new diagnostic approach to IBD using blood plasma or saliva in humans by NIR spectroscopy and Aquaphotomics^17^. This study showed that patients had a history of contact with livestock, engaging in agricultural activities, using non-piped water, consuming unpasteurized dairy products, having a family history of IBD, or living in rural areas. These findings suggest that contact with contaminated livestock -probably with MAP- and their products, including milk, meat, and their by-products, as well as exposure to contaminated manure and water, may significantly impact the IBD rate^17^. Some of the candidate genes identified across studies overlap with those found in Crohn’s disease and tuberculosis including; Solute carrier family 11 member of one gene (SLC11A1), Nucleotide-binding-oligomerization domain-containing 2 (NOD2), Major histocompatibility complex type II (MHC-II), and Toll-like receptor (TLR) genes^18^.

Using the appropriate tests and methods to detect MAP infection and prevent its spread in the shortest possible time is very critical^19^. The diagnostic tests for MAP infection are primarily infection detection tests and tests to identify the host’s immune response to the bacterium. Two techniques of bacterial culture and tracking its molecular component by polymerase chain reaction technique (PCR) are usually used to diagnose MAP strains^20^. The molecular techniques of enzyme-linked immunosorbent assay (ELISA), complement fixation test (CFT), and agar gel immunodiffusion (AGID) have been frequently used to assess the host immune response^21^. Still, the level of accuracy and sensitivity of each of these techniques are very different, for example, the sensitivity of the milk ELISA test is 21 to 61%, the blood plasma ELISA test is 7–94%, Fecal PCR is 4 to100% and fecal culture is 20 to74%^22^.

It should be noted that the mentioned molecular tests are very effective in early screening and tracking and that histopathological studies provide the possibility of definitive and accurate diagnosis of JD, especially its tissue effects. According to studies, while the ELISA test is the most cost-effective tool for the detection of anti-MAP antibodies, the PCR test or feces culture (FC) testing is preferred to reduce the prevalence, and both are assumed to be more sensitive for low-shedding animals (Robins et al., 2015; Smith, et al., 2017). Others believe combining ELISA and PCR and their serial interpretation will be the most cost-effective possible method (Aly et al., 2012).

As a result, the lack of early and accurate diagnostic tests and MAP’s inherent resistance to antibiotics and disinfectants have made JD infection control very difficult, turning Johne’s disease into a global challenge. Therefore, developing an accurate diagnostic method to distinguish healthy from infected animals based on the disease agent that produces antibodies and the ability to show the animal’s state at each stage of the disease is a global necessity.

Nowadays, using modern physical methods in disease diagnosis is preferable to chemical methods due to their higher accuracy, non-invasiveness, cost and time effectiveness, and fewer side effects. Some research was based on modern physical methods to diagnose JD.

NIR spectroscopy, as one of the physical and non-invasive methods, without any sample preparation or chemical pollution, with high accuracy and based on molecular bond vibration measuring, by establishing the interaction of electromagnetic waves with biological materials has been widely used to quantify crop nutrient composition and for quality control across the food industry and pharmaceutical products in the last 50 years. Water is cited as one of the disadvantages of NIR spectroscopy in aqueous systems because it could alter sample spectra, hide absorbance bands, and shift absorbance bands. Another advantage of NIR is that it detects amounts smaller than 500ppm in solution. For this reason, in the last decade, the application of this method, including structural analysis of water, has grown significantly. The Aquaphotomics approach to analyzing water and aqueous systems such as plasma, serum, urine, and milk spectra provides a unique opportunity to describe the complex state of water using its multidimensional NIR spectra^23^.

Aquaphotomics is a novel scientific discipline founded by Professor Roumiana Tsenkova in 2005, involving the study of water and aqueous systems, using light–water interaction to extract information about the structure of water, composed of many different water molecular information by using absorbance bonds, which found to be huge source of information on the subject of the structural and related function properties of aqueous system. Aquaphotomics is a complementary “omics” discipline with the large-scale, comprehensive studies of water as a “collective matter and energy mirror” of the rest of the aqueous system that has memory and conciseness to detect perturbation and change the arrangement to keep biosystem sustainability. While genomics studies genes, proteomics - proteins, glycomics- carbohydrates, and lipidomics—lipids, aquaphotomics explores the roles, relationships and functions of the water- an equally important biomolecule and one of nature’s fundamental building blocks. Water, a natural matrix of any aqueous or biological system, changes its absorbance pattern every time it adapts to a physical or chemical change in the system itself or its environment. Small quantities or structural modifications of other molecules are present in the aqueous system, so the information is extracted about not only water structure but also other components present in water or the state of the system as a whole. Aquaphotomics changes the treatment of water in spectroscopy to an opportunity to achieve much valuable information and, in this role, water acts like a biosensor and amplifier, especially for low concentration amounts, and detects them and their changes^24,25^.

Aquaphotomics which uses the high sensitivity of water’s hydrogen bonds, is, in accordance with any other water system, a dynamic arrangement of a network of water molecules that are hydrogen-bonded with other constituents and are affected by perturbations like infection. Any internal or external perturbation of the aqueous system results in changes in water molecular conformations, which in turn produce changes in the corresponding NIR spectra at their respective water absorbance bands. In the range 1300–1600 nm, 12 water absorbance bands are more important. Water spectral pattern as a holistic biomarker, which relates certain water structures with functionalities of the respective biological system and thus opening new directions to ward non-destructive quality monitoring applications and non-invasive bio-diagnosis^24,25^.

NIR spectroscopy and Aquaphotomics models need chemical methods as a reference test for calibration and then can predict the samples by models. In this study, blood plasma and milk ELISA tests were selected for reference tests, separately. For increasing accuracy, samples were selected according to the same three consequent results of tests. Applying two chemometrics methods for the evaluation of one aspect of the experimental study demonstrates the stability of the applied methodology, namely, consistency in results^24^. In this way, pre-processing and three PCA methods were used as an unsupervised method in the first one followed by QDA and SVM, as supervised methods to distinguish between healthy and infected dairy cattle by Paratuberculosis, the consistency in our results in a total range 1300–1600 nm and then in 12 water absorbance bands.

Aquaphotomics, applied for discrimination of healthy and mastitic animals based on the spectra of urine, blood, and milk of dairy cows^26–29^. Estrus detection in cow and panda^30–32^, discrimination of different bacteria strains^33–36^, pneumonia in dairy calves^37^ and detection of type2 diabetic^38^, Paratuberculosis diagnosis by blood plasma^11^and saliva^39^.

NIR spectroscopy and Aquaphotomics combined with chemometrics-based multivariate analysis (MVA) may be able to identify and discriminate the biochemical profile of milk associated with Paratuberculosis infection and monitor the water molecules of milk response to perturbation of this infection in dairy cattle. According to the function of the collective mirror, water, all of the changes in biomolecules, antibodies, or MAP in Johne’s disease that today measure separately may be monitored by water absorbance bands accurately.

The objectives of this study were to use milk for diagnosis of Paratuberculosis by NIR spectroscopy and supervised methods, then use water absorbance bands of milk for biomonitoring and to discover water spectral changes of milk associated with diagnosing Paratuberculosis using Aquaphotomics. The current diagnostic methods for Paratuberculosis require detecting MAP through feces culture (FC), PCR test, or measuring an adequate level of antibodies through ELISA test, but not accurate in low concentration. Aquaphotomics can detect the low concentration of antibodies or other changes in biomolecules by infection of Paratuberculosis perturbation.

**Fig. 1:**
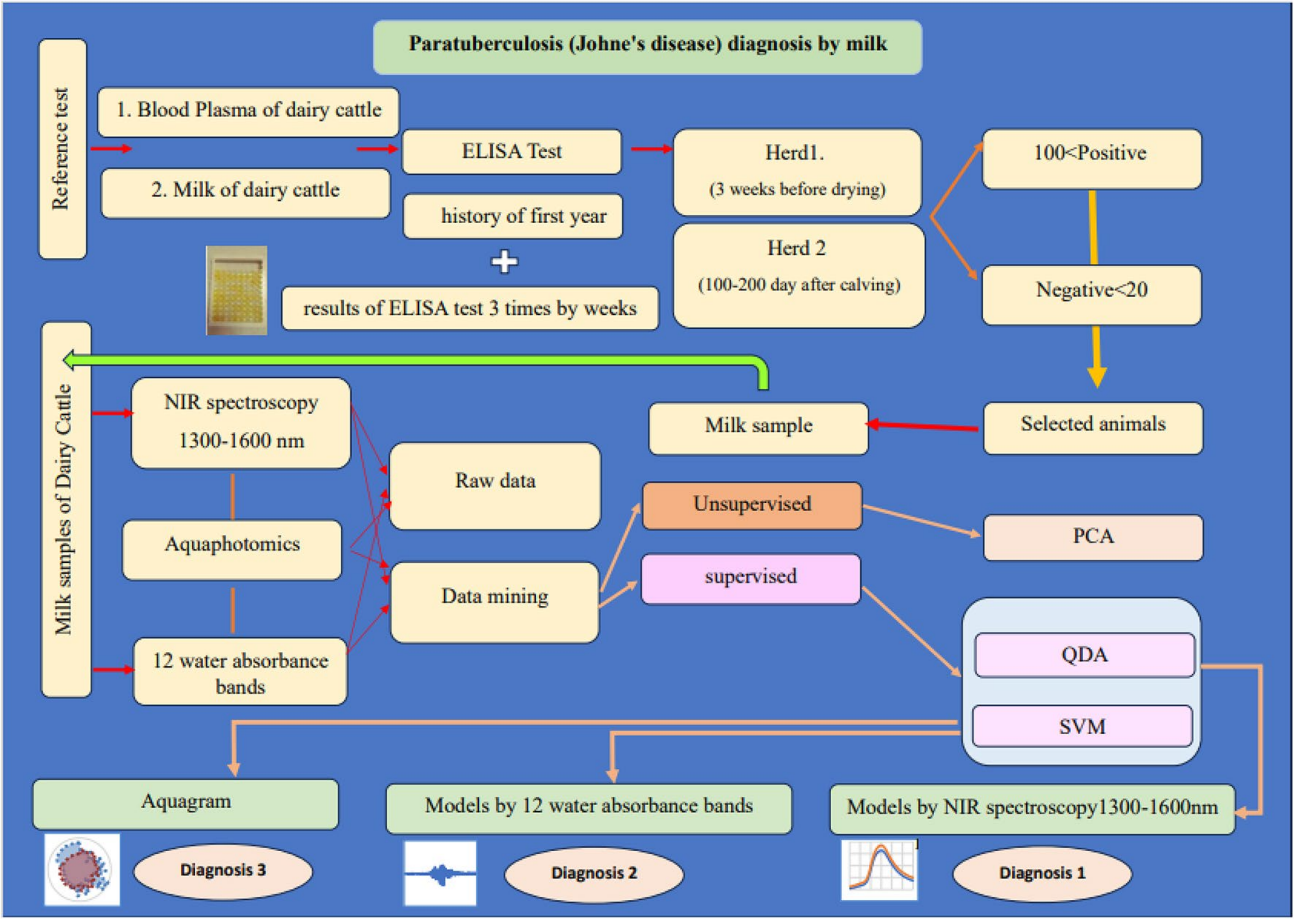
The brief of research “Milk a silver key to diagnosis of Partuberculosis in dairy cattle using Near- infrared spectroscopy and Aquaphotomics”. *PCA* principal component analysis, *QDA* quadratic discriminate analysis, *SVM* support vector machine.

The long-term goal of this research group is to create a diagnostic strategy for detection of dairy cattle in the 4 stages of Johne’s disease and to introduce a non-invasive, accurate, and fast diagnostic method in vivo, which will contribute to the sustainability of food immunity and global health. Figure 1 shows the brief of this study.

## Results

### Raw absorbance spectra of milk

**Fig. 2:**
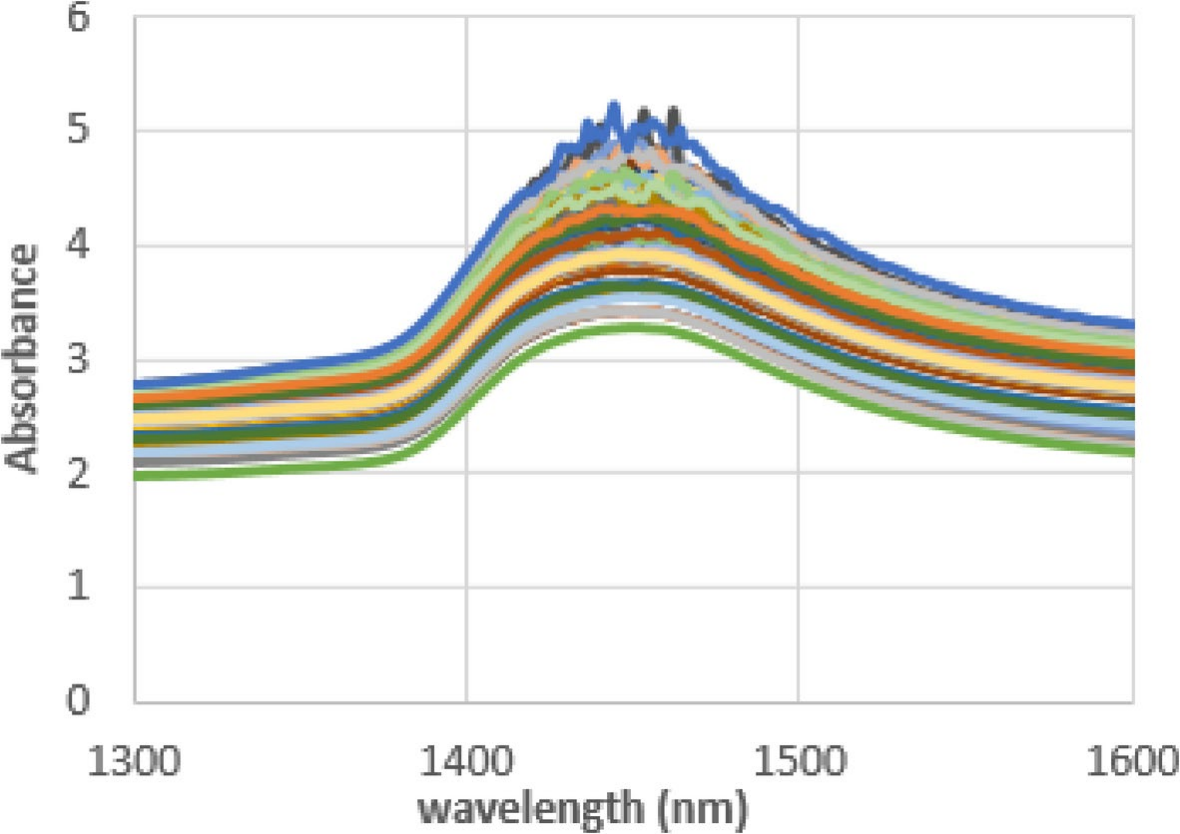
Milk raw data absorbance spectra of dairy cattle in the range of 1300–1600 nm.

Raw NIR absorbance spectra in the analyzed 1300–1600 nm range are shown in (Fig. 2). In this spectral region, these spectra appear identical, with the main feature being a dominant absorbance band around 1450 nm attributed to the first overtone of OH stretching vibration^40^. Since bovine milk contains 87% water^41^, the spectra of milk are similar to the water spectra.

**Fig. 3:**
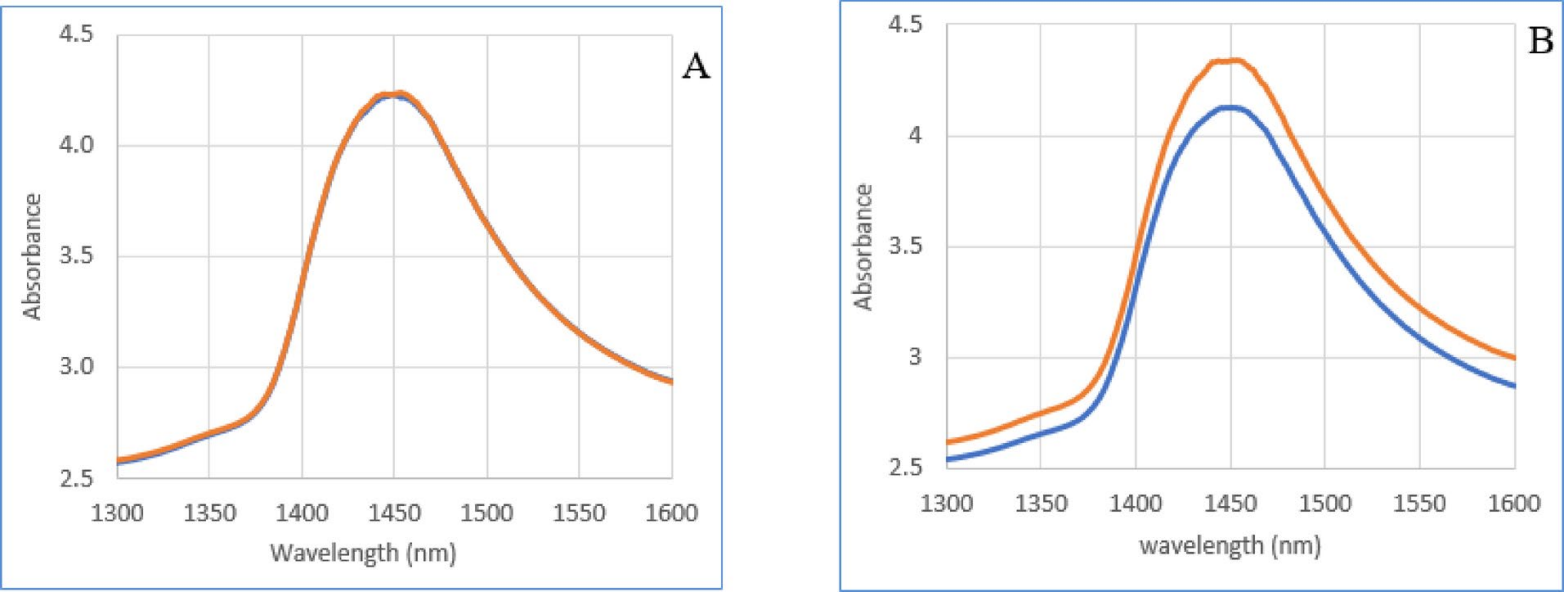
The mean absorption spectra of milk. (**A**) It based on the results of the blood plasma ELISA test as a reference test. (**B**) It based on the results of the milk ELISA test as a reference test. The negative group is blue and positive group is red.

The means of spectra and spectral subtraction were evaluated in two groups, negative and positive. This was followed by a regression coefficient in PLS-DA analysis to enhance the subtle changes at specific water absorbance bands in the spectra of milk samples^24^. The raw data mean of milk for each group, according to blood plasma ELISA test as a reference test, is not interested only in 1420 to 1470 nm (Fig. 3A). The difference between two groups was evident based on the milk ELISA test as a reference. The positive group had higher absorption than the negative group, which indicate that the amount of water in the milk of infected animals was higher (Fig. 3B).

**Fig. 4:**
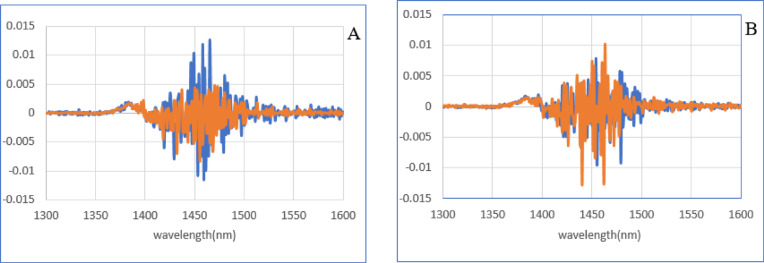
The mean of second derivative of milk spectra. (**A**) It based on the results of the blood plasma ELISA test as a reference test. (**B**) It based on the results of the milk ELISA test as a reference test. The negative group is blue and positive group is red.

**Fig. 5:**
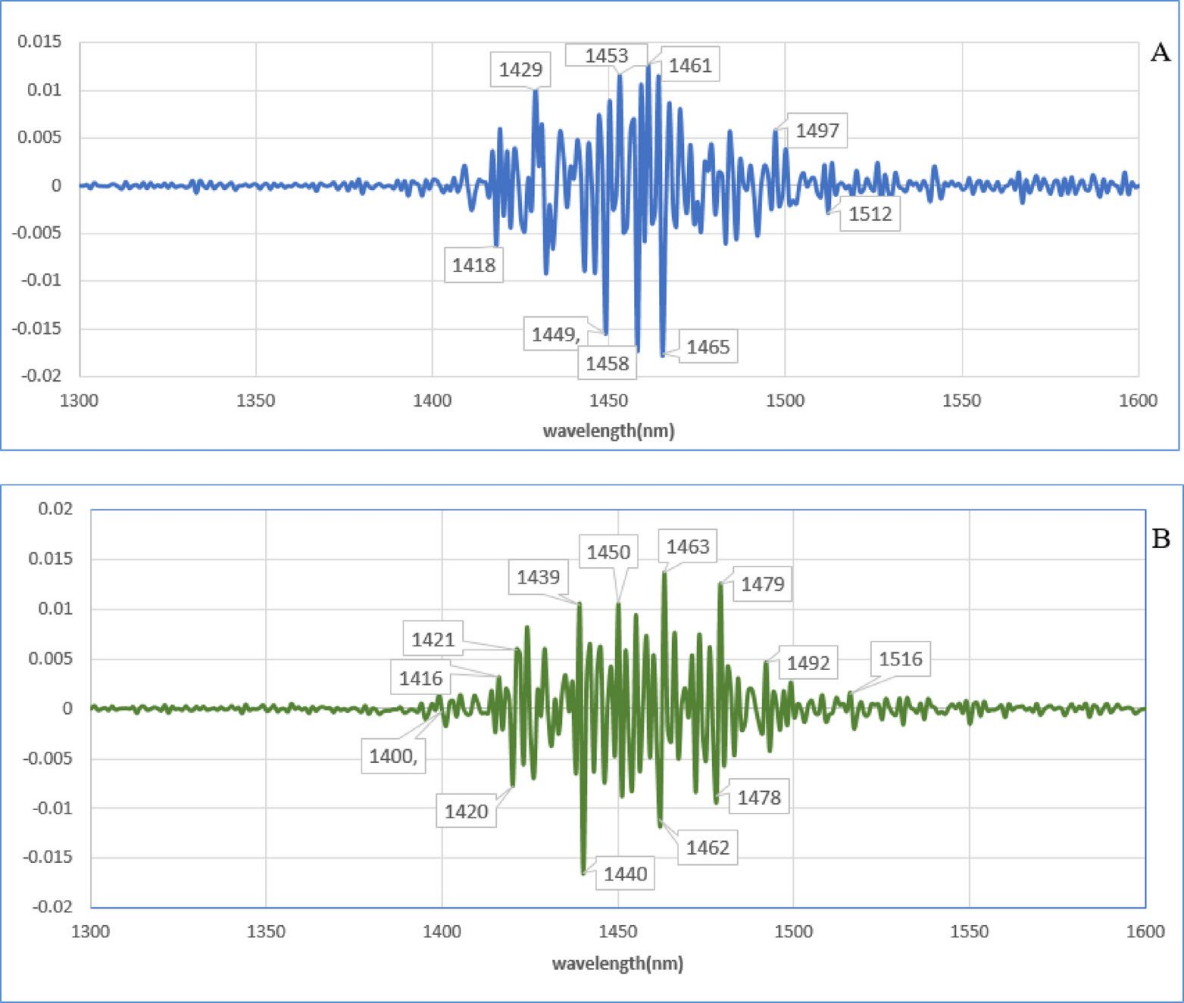
The subtracted of the means of negative and positive groups from the total mean in second derivative of milk spectra. (**A**) It based on the results of the blood plasma ELISA test as a reference test. (**B**) It based on the results of the milk ELISA test as a reference test.

The means of the second derivation of data in the negative and positive groups were compared to emphasize the subtle differences (Fig. 4). Then the second derivation of data in each group was subtracted from the total average of all spectra according to the blood plasma ELISA test results (Fig. 5A) and according to the milk ELISA test results, as a reference test (Fig. 5B). This spectral subtraction enhanced the differences between the two groups in the second derivation data, and the most considerable differences are in the regions around 1400–1500 nm, according to the C5 to C11 water absorbance band.

About the effect of MAP and IBD infection, the healthy group and patients were asked about contact with livestock, agricultural activities, non-piped water, non-pasteurized dairy products, family history, place of residence in childhood and now, and occupation. Out of a total of 10 patients with IBD, 30% had a family history, 70% now resided in cities, and 70% spent their childhood years in villages. 90% of the case group uses piped water now, and 70% used non-piped water when they were children. 50% of the case group used unpasteurized milk and dairy products during childhood and now. 20% were non-slaughterhouse meat consumers; 50% had agriculture activity; 40% were in contact with livestock; 60% were in contact with soil and manure; 10% were employed in slaughterhouses; and finally, 80% were in contact with milk and dairy product production. These results can confirm the effect of contact with contaminated livestock and its products, including milk, meat, and their products, and contaminated manure and water on IBD rate.

### PCA- exploratory analysis of paratuberculosis effects on spectra of milk

**Fig. 6:**
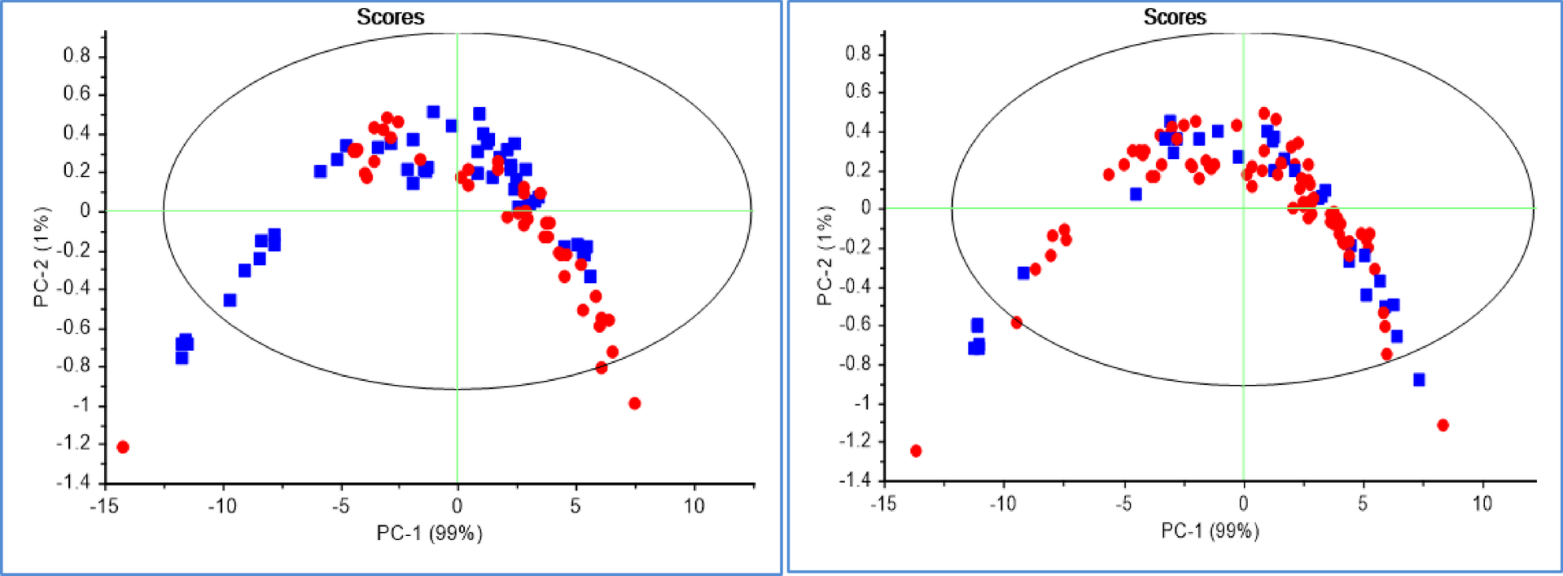
Scores plot of PCA milk spectra. (**A**) based on the results of the blood plasma ELISA test as a reference test. (**B**) based on the results of the milk ELISA test as a reference test. The negative group is blue and positive group is red.

The PCA is an unsupervised multivariate analysis that reduces the dimensionality of data sets, explains the variation in the data by ignoring the data label, helps in the detection of patterns in the spectral behavior, and finds excluded data (Ghasemi et al., 2013). The results of PCA were presented as scores and loading plots. In the PCA score plot, the similarity and differences in chemical complexes that contain OH, CH, and NH bonds interact with NIR light in transformed spectra of bovine milk in 1300–1600 nm range can be observed in Fig. 6. Based on this, by performing PCA analysis on all data, in according to with the established criteria, the samples that were outside the Hotling T2 ellipse with an accuracy of 95% were recognized as outliers and were removed from the calculated data category. From the total of 110 independent spectra obtained from both negative and positive groups, 16 spectra were recognized as outliers and removed from the data set. Subsequent analyses were performed on the remaining 94 samples. Of course, it should be noted that milk has many compounds with various amounts, and these amounts will be different depending on the animal’s physiological stage and herd management. Here, due to the use of data from two livestock farms and two different physiological stages, the outlier data increased compared to the plasma ^10^. Subsequent analyses were performed on the remaining 94 spectra. According to the scores chart of the raw data, the first two principal components describe all the variance of the total data, so that PC1 is 99% and PC2 is 1%. Figure 5 shows the distribution of samples in both negative and positive classes of raw data obtained in PC1-PC2 space.

As can be seen, despite some admixture between healthy and infected samples in the PC1-PC2 space, the two groups were separated from each other to the acceptable extent expected from a non-learning analysis such as PCA. In PCA models, according to the blood plasma ELISA as a reference test, while the negative samples tended to be placed in the first, second, and fourth quadrants of PC1-PC2 space, the positive samples were more scattered in the first to fourth quadrants of this space (Fig. 6A). In PCA models, according to the milk ELISA test as a reference test, while the negative samples tended to be placed in the first, second, and third quadrants of the PC1-PC2 space, the positive samples were more scattered in the first, second and, fourth quadrants of this space. The grouping in this part is clearer (Fig. 6B). According to the descriptive variance by the first two components and the separation algorithm, it is expected that the discernment analysis such as QDA can better separate these two groups of samples. The PCA analysis was performed also with various pre-processing techniques.

**Fig. 7:**
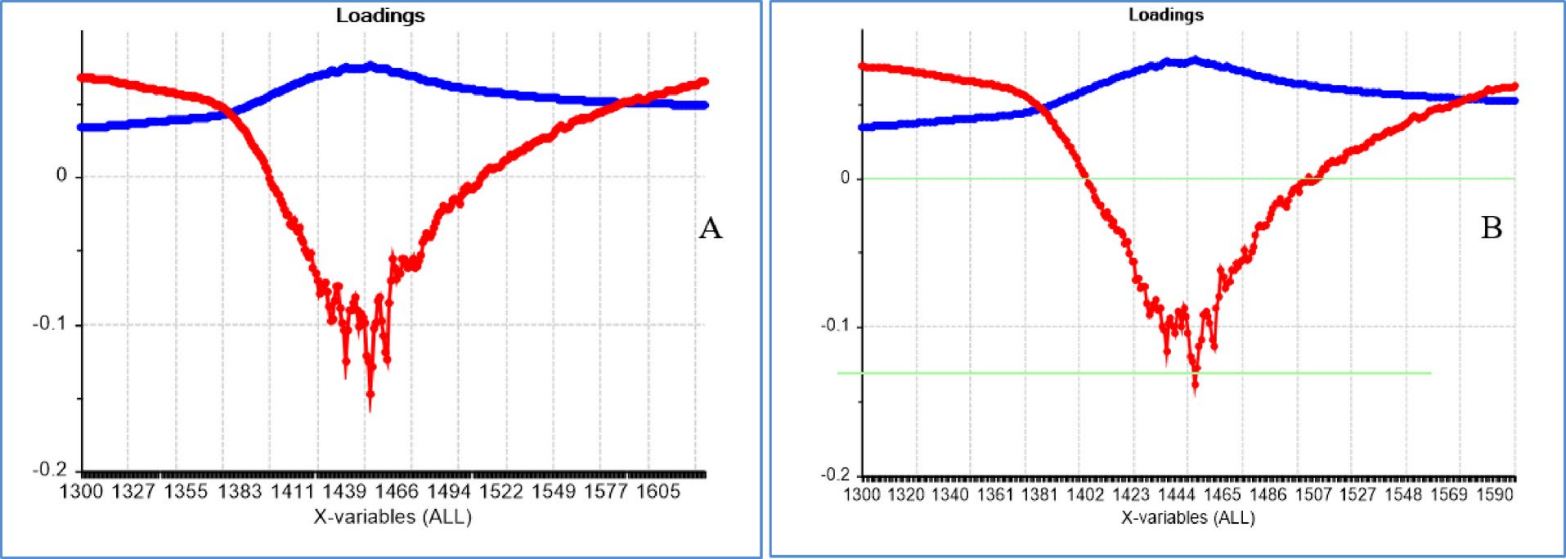
loading plot of PCA milk spectra. (**A**) based on the results of the blood plasma ELISA test as a reference test. (**B**) based on the results of the milk ELISA test a reference test. The PC1 is blue and PC2 is red.

The PCA loadings revealed the dominant peaks influencing the trends in the score plot (Fig. 7), which were related to the OH in water bonds. PC1 represented the most variation in the data (99%) and PC2 (1%).

### QDA analysis for detection of dairy cattle response to paratuberculosis of milk

One type of discriminant analysis is quadratic discriminant analysis (QDA). When each group’s variability does not have the same structure (unequal covariance matrix) and the curve shape separating groups is not linear, QDA provides a good classification model. The QDA models of milk according to the blood plasma ELISA test as the reference test, in internal validation, in the range of 1300–1600 nm for the total wavelength of raw data in negative and positive groups for specificity and sensitivity was 100%, and 97% and for calibration data was 100%, and 100%, respectively. External validation separates the positive and negative groups based on 82% total accuracy, 100% sensitivity, and 20% specificity. In internal validation, QDA in WAMACs in milk has 100 and 100% sensitivity and 100 and 100% specificity for full data and calibration, respectively. External validation was 82% total accuracy, 100% sensitivity, and 20% specificity in Paratuberculosis detection. Models of WAMACs had more accuracy in full data, and the same accuracy in calibration and test data (Table 1).

**Table 1:** The results of supervised methods (QDA, SVM) of milk data to detect positive and negative groups of paratuberculosis in the range of 1300–1600 nm and 12water absorbance bands (WAMACs) according to the results of blood plasma and milk ELISA tests as the reference tests.

Model	Area	range	Predicted model		
			Full data–internal validation (%)	Calibration–internal validation (%)	Test–external validation (%)
QDA (milk with blood plasma ELISA test as a reference test)	1300–1600 nm	Negative	100	100	20
		Positive	97	100	100
		Total Accuracy	98	100	82
		Pretreatment	Raw data- 1st derivation- spectroscopic	Raw data- smoothing-1st derivation & 2th derivation- Baseline- Normalized	2th derivation
	12 water absorbance bands	Negative	100	100	20
		Positive	100	100	100
		Total accuracy	100	100	82
		Pretreatment	Spectroscopic	Raw data- 2th derivation- baseline- smoothing- normalize-	2th derivation
QDA (milk with milk ELISA test as a reference test)	1300–1600 nm	Negative	90	95	90
		Positive	100	100	54.5
		Total accuracy	95	97	71
		PC	20	19	19
		Pretreatment	Spectroscopic	Spectroscopic	Smoothing
	12 water absorbance bands	Negative	90	95	90
		Positive	100	100	54.5
		Total accuracy	95	97	71
		PC	20	19	19
		Pretreatment	Spectroscopic	Spectroscopic	Smoothing
SVM (milk with blood plasma ELISA test as a reference test)	1300–1600 nm	Negative	100	100	0
		Positive	100	100	100
		Total accuracy	100	100	77
		Pretreatment	Raw data -all pretreatment	Raw data -all pretreatment	Raw data -all pretreatment
		R^2^ - RSME	1–0.1	1–0.05	
	12 water absorbance bands	Negative	100	100	20
		Positive	100	100	100
		Total accuracy	100	100	82
		Pretreatment	Raw data -all pretreatment	Raw data -all pretreatment	Spectroscopic
		R^2^ - RSME	1–0.05	1–0.05	99–0.05
SVM (Milk with milk ELISA test as a reference test)	1300–1600 nm	Negative	100	100	90
		Positive	100	100	73
		Total accuracy	100	100	81
		Pretreatment	Raw data- all pretreatment	Raw data- all pretreatment	Baseline
		R^2^- RMSC	1–0.05	1–0.05	0.99−0.04
	12 water absorbance bands	Negative	100	100	80
		Positive	100	100	82
		Total accuracy	100	100	81
		Pretreatment	Raw data- all pretreatment	Raw data- all pretreatment	Baseline
		R^2^- RMSC	1–0.05	1–0.05	0.99−0.04

The QDA models of milk according to the milk ELISA test as a` reference test, in internal validation, in the range of 1300–1600 nm for the total wavelength of raw data in negative and positive groups was 90%, and 100%, and for calibration data was 95%, and 100%, respectively, for specificity and sensitivity. External validation separates the positive and negative groups based on 71.4% total accuracy, 54.5% sensitivity, and 90% specificity. In internal validation, QDA in WAMACs in milk has 90% and 100% sensitivity and 95 and 100% specificity for full data and calibration, respectively. External validation was 71.4% total accuracy, 54.5% sensitivity, and 90% specificity in Paratuberculosis detection. Models of WAMACs had the same accuracy in full data and calibration in internal validation and test data in external validation (Table 1).

### SVM analysis for detection of cattle response to paratuberculosis of milk

The SVM has been used to detect Paratuberculosis and health groups as a powerful supervised method. The SVM analysis of milk data, according to the blood plasma ELISA test as a reference test, the prediction equation obtained from the raw data, and all pretreatments, which were the result of internal validation of full data and calibration, contained 100% specificity with 100% sensitivity. The total accuracy, sensitivity, and specificity for external validation were 77.3%, 100%, and 0%, respectively. These models had a high coefficient of determination (R^2^) of 100% and a low root mean square error of prediction (RMSEP) of 0.05–0.1%. This time, SVM was performed based on Aquaphotomics. In comparison to the data obtained from SVM by water absorbance bands (WAMACs) with the results obtained from all wavelengths in the range of 1300–1600 nm, in internal validation models for full data and calibration were the same, with 100% sensitivity and 100% specificity. The coefficient of determination, R²=100%, and RMSEP = 0.05–0.1 were obtained in separating the negative and positive groups. External validation shows that the model with raw data or all pretreatment achieved 82% total accuracy, 100% sensitivity, and 20% specificity in separating positive and negative groups for Paratuberculosis. This increase in total accuracy, sensitivity, and specificity demonstrates the high contribution and key role of water absorbance bands in the results of the SVM models in the 1300–1600 nm range according to the blood plasma ELISA test results as a reference test.

The SVM analysis of milk data, according to the milk ELISA test as a reference test, the prediction equation obtained from the raw data, and all pretreatments, which were the result of internal validation of full data and calibration, contained 100% specificity with 100% sensitivity. The total accuracy, sensitivity, and specificity for external validation were 81, 73, and 90%, respectively. These models had a high coefficient of determination (R^2^) of 100% and a low root mean square error of prediction (RMSEP) of 0.05–0.1%. This time, SVM was performed based on Aquaphotomics. In comparison to the data obtained from SVM by water absorbance bands (WAMACs) with the results obtained from all wavelengths in the range of 1300–1600 nm, internal validation models for full data and calibration were the same, with 100% sensitivity and 100% specificity. The coefficient of determination, R²=100%, and RMSEP = 0.05–0.1 were obtained in separating the negative and positive groups. External validation shows that the model with baseline pretreatment achieved 82% total accuracy, 100% sensitivity and 20% specificity in separating positive and negative groups for Paratuberculosis. This increase in sensitivity with equal accuracy demonstrates the high contribution and key role of water absorbance bands in the results of the SVM models in the 1300–1600 nm range according to the milk ELISA test results as a reference test.

These findings suggested that biochemical changes related to antibody amount in milk as a result of the dairy cattle response to MAP infection in Paratuberculosis could be accurately detected and classified using NIR spectroscopy and Aquaphotomics according to the same three concequent blood plasma ELISA test results or milk ELISA test results as a reference test. The SVM models had 100% accuracy full data and calibration data in internal validation. Decrease in accuracy in external validation may be due to the low number of samples (Table 1).

### Aquagrams

Finally, the difference in the water structure in the milk of the negative and positive groups according to the reference tests, containing the blood plasma ELISA test and the milk ELISA test were determined. It can be investigated using the aquagram of the active water absorbance bands created during the disturbance, which is Paratuberculosis in this case. The aquagrams display normalized absorbance values from MSC pretreatment at water absorbance bands on the axes originating from the center of the graph to identify the water absorbance bands that responded strongly to Paratuberculosis. WAMACS absorbance values were used for axes. By comparing the aquagrams for the positive and negative groups, the relationship between two groups of infected and healthy individuals with WASP was estimated.

Based on the results of the mean spectrum difference, all WAMACS wavelengths distinguish between positive and negative groups and are thus used in drawing the aquagram.

**Fig. 8:**
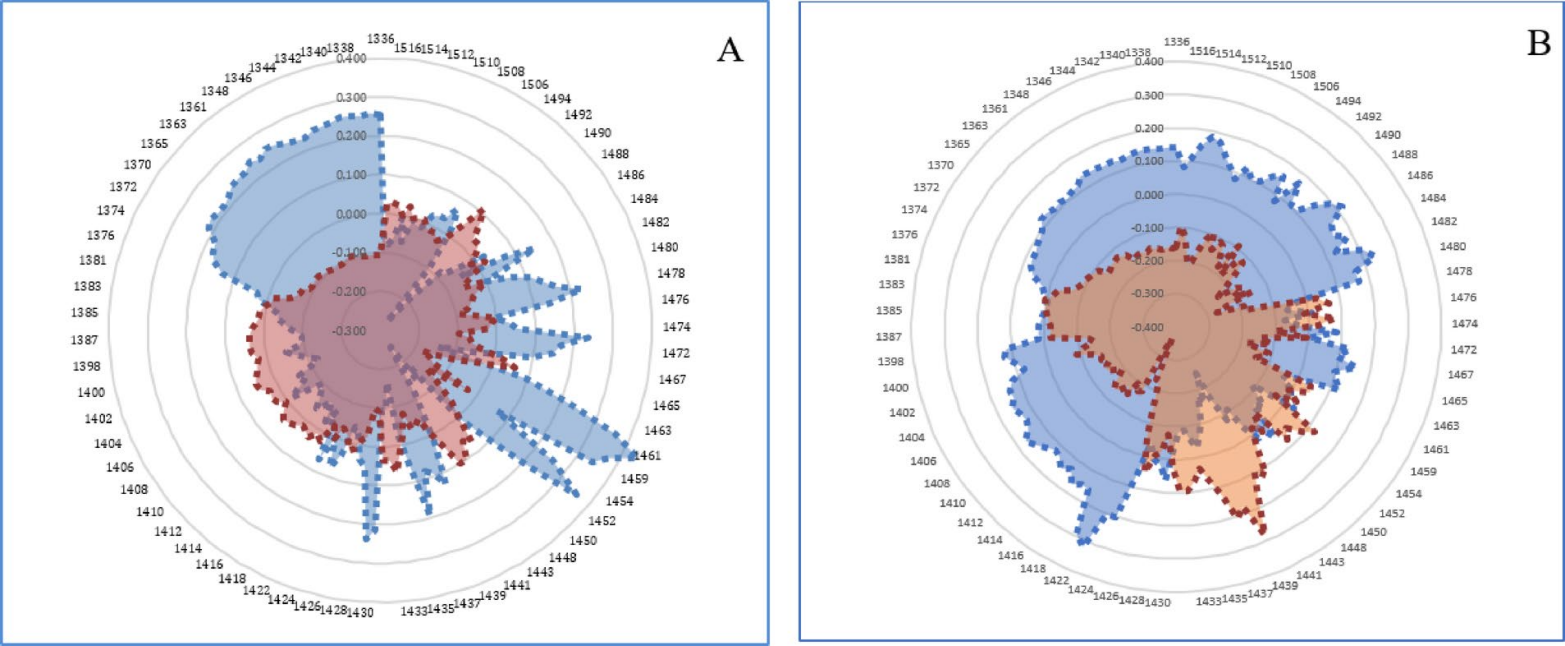
Aquagram of milk samples in two groups, healthy and infected, using MSC pre-processing and the standardized data of the 12 water absorbance bands of in the range of 1300–1600 nm. (**A**) based on the results of the blood plasma ELISA test. (**B**) based on the results of the milk ELISA test as a reference test. Healthy group (blue) and Infected group (red).

In aquagram of water absorbance bands of milk according to the blood plasma ELISA test, as a reference test, active water absorbance bands in the negative class include C1: water with asymmetric stretching vibration, C2: water solvent shell, and C3: water bands with symmetric and asymmetric stretching vibration, C6: have free OH bands and hydrated water, C7: bands with a hydrogen bond, C8: water solvation shell and, C9: Water molecules with 2 hydrogen bonds had more absorbance than positive groups. In addition, water absorbance bands C4: Hydrated superoxide clusters and symmetrical stretching vibration, C5: containing free water, and C11 with four hydrogen bonds, in the positive group had more absorbance than the negative groups (Fig. 8A).

In other words, the effect of changes in antibody of blood plasma causing Paratuberculosis can be observed in Water Absorbance spectral patterns (WASPs) of milk, where the water with hydrated superoxide clusters and symmetrical stretching vibration, free water proportion and, having four hydrogen bonds in the milk is significantly reduced (Fig. 8B).

In aquagram of water absorbance bands of milk according to the milk ELISA test as a reference test, the absorbance in active water absorbance bands in the positive group include C1: water with asymmetric stretching vibration, C2: water solvent shell, C5: containing free water, C6: have free OH bands and hydrated water, C11 with four hydrogen bonds, C12 with stronger bonds decreased and C4: Hydrated superoxide clusters and symmetrical stretching vibration and C7: bands with a hydrogen bond increased.

## Discussion

Paratuberculosis causes changes in the amount of antibodies in dairy cattle’s blood plasma and milk. An ELISA kit can detect these changes. Due to the characteristics of Paratuberculosis and the lack of 100% diagnostic accuracy of this kit, animals with a history of Paratuberculosis were tested for three consecutive weeks. However, in the blood plasma, and ELISA test results, as a reference, animals with three similar test results and an index of less than 20 were considered healthy or negative. Animals with an index greater than 100 were classified as infected or positive. So, the two groups were chosen based on the results of three consecutive weekly ELISA blood plasma tests in dairy cattle: a healthy group with an index of < 20 and an infected group with an index of > 100. Another model was developed according to the milk ELISA test results, as a reference test.

NIR spectrum profile was used to develop two classification models based on the antibody index in plasma and milk to monitor the biochemical changes caused by Paratuberculosis in the blood plasma, milk, and particularly in the structure of the water molecules that make them. These models diagnosed Paratuberculosis and distinguished healthy and infected samples with 100% total accuracy, sensitivity, and specificity. Finally, the aquagram was obtained, which shows the changes in the 12 water bands in healthy and infected dairy cattle.

First, datasets were created according to the blood plasma ELISA test and milk ELISA test as the reference tests separately. The null hypothesis (no biological signature can distinguish samples from healthy and infected groups) was tested by NIR spectroscopy and Aquaphotomics approach to the unsupervised and supervised analysis in two ranges, 1300–1600 nm, and only in 12 water absorbance bands in this range.

NIR spectroscopy can provide information on the chemical and physical composition of raw materials such as milk, particularly on portable instruments that can be used directly on the dairy farm^42^. Additionally, NIR spectroscopy was used to detect genetically modified organisms. Aquaphotomics is a novel field that could be explored for monitoring them^43^.

The physiological and metabolic changes are the foundation for NIR spectroscopy profiling to distinguish between healthy and infected animals. It can be used to detect and monitor infections in dairy cattle. The profile of NIR spectra reflects the animal’s immune response to Johne’s disease agent by applying multivariate analysis and the Aquaphotomics approach based on the biochemical changes that caused the difference between the blood plasma of healthy and infected animals like other research^28,30,44^. In 2012, Mycobacterium Tuberculosis was detected by infrared and near-infrared spectroscopy^45^.

This study analyzed the milk spectra using PCA, QDA, and SVM methods according to the blood plasma and the milk ELISA test as the reference tests for calibration. These analyses reveal information about biochemical imbalances caused by diseases. These effects cause the changes in the structure of the water in the milk, characterized by the reuse of PCA, QDA, and SVM methods in the range of 12 water absorbance bands and can be seen in the aquagram.

Since the accuracy of the milk ELISA test is lower than that of blood plasma, the diagnostic results of these tests as the reference test, were not the same in the studied animals. Therefore, the models were developed based on the results of each test and their diagnostic accuracy was reported separately. Thus, using the same response of three blood ELISA tests as a reference test and selecting calibration animals based on it, and using a milk sample, which is easier than a blood sample, as the examined sample, leads to the development of more accurate diagnostic models than the model Calibrations were based on milk ELISA results and milk samples.

The raw data spectrum samples and the average of the negative and positive groups followed the water spectral pattern, with a peak at 1450 nm, which confirms the high proportion of water in milk compounds.

In PCA models, according to the blood plasma ELISA as a reference test, while the negative samples tended to be placed in the first, second, and fourth quadrants of PC1-PC2 space, the positive samples were more scattered in the first to fourth quadrants of this space. In PCA models according to the milk ELISA test as a reference test, while the negative samples tended to be placed in the first, second, and third quadrants of the PC1-PC2 space, the positive samples were more scattered in the first, second, and, fourth quadrants of this space. The grouping in this part is clearer.

The QDA models of milk according to the blood plasma ELISA test as the reference test, in internal validation, in the range of 1300–1600 nm for the total wavelength of raw data in negative and positive groups was 100%, and 97%, and for calibration data was 100%, and 100%, respectively, for specificity and sensitivity. External validation had 82% total accuracy, 100% sensitivity, and 20% specificity. In internal validation, QDA in WAMACs in milk has 100% and 100% sensitivity and 100 and 100% specificity for full data and calibration, respectively. External validation was 82% total accuracy, 100% sensitivity, and 20% specificity in Paratuberculosis detection. Models of WAMACs had more accuracy in full data and the same accuracy in calibration and test data.

The QDA models of milk according to the milk ELISA test as the reference test, in internal validation, in the range of 1300–1600 nm for the total wavelength of raw data in negative and positive groups was 90%, and 100%, and for calibration data was 95%, and 100%, respectively, for specificity and sensitivity. External validation separates the positive and negative groups based on 71.4% total accuracy, 54.5% sensitivity, and 90% specificity. In internal validation, QDA in WAMACs in milk has 90 and 100% sensitivity and 95 and 100% specificity for full data and calibration, respectively. External validation was 71.4% total accuracy, 54.5% sensitivity, and 90% specificity in Paratuberculosis detection. Models of WAMACs had the same accuracy in full data and calibration in internal validation and test data in external validation.

The study on the diagnosis of Paratuberculosis by QDA models showed blood plasma NIR spectroscopy and Aquaphotomics had 100% specificity, 98, and 97% sensitivity in internal validation in full data and calibration, respectively. In external validation, had 100% specificity and sensitivity. The results in 1300–1600 nm and 12 water absorbance bands are the same^11^. Another study on the diagnosis of Paratuberculosis by QDA models showed saliva NIR spectroscopy and Aquaphotomics had 100% specificity, 98% sensitivity in internal validation, and 67% specificity and 100% sensitivity in external validation. The results in 1300–1600 nm and 12 water absorbance bands are the same.

The study on the diagnosis of Paratuberculosis by SVM models showed that blood plasma NIR spectroscopy and Aquaphotomics had 97, 99% specificity, and 100% sensitivity in internal validation in full data and calibration, respectively. In external validation, had 100% specificity and sensitivity. The models according to the 12 water absorbance bands had 100% specificity and 98, and 97% sensitivity in full data and calibration data, respectively^11^. External validation had 100% specificity and sensitivity. Another study on the diagnosis of Paratuberculosis by SVM models showed saliva NIR spectroscopy and Aquaphotomics had 100% specificity, 98% sensitivity in internal validation, and 83% specificity and 93% sensitivity in external validation. The results in 1300–1600 nm and 12 water absorbance bands are the same.

The results of these studies and the current study showed the power of NIR spectroscopy and Aquaphotomics to diagnose Paratuberculosis by blood plasma, saliva, and milk by QDA and SVM models in 1300–1600 nm or only 12 water absorbance bands by 100% specificity, and sensitivity.

Early diagnosis of type 2 diabetes based on NIR Spectroscopy and SVM model with Aquaphotomics approach showed 97.22% accuracy, and the specificity and sensitivity were 95.65 and 100%, respectively with first derivative pretreatment. This study shows that the combination of NIR spectroscopy with Aquaphotomics is an accurate, rapid, and effective method to develop a model for the early diagnosis of diabetes^38^.

NIR spectroscopy was used in 2006 as a new approach to diagnose Paratuberculosis in dairy cattle. In this study, NIR spectroscopy and artificial neural network (ANN) method, with fecal culture and serum test ELISA were used as reference test. In this research, NIR spectroscopy was used as an indirect test for Paratuberculosis infection in cattle. However, it was not clear which parameters or substances present in serum can be discriminates by the NIR spectroscopy^46^. The current study showed that the water absorbance bands play the critical role in distinguishing groups from each other.

The total accuracy of using milk NIR spectroscopy to diagnose Johne’s disease in dairy cattle with a 95% confidence margin is 100% in full data, calibration, and test, respectively, which is comparable to the sensitivity of current diagnostic models, such as ELISA test (7–94%), fiscal culture (20–74%), and PCR (4–100%).

Invite 100% accuracy, sensitivity, and specificity to detect Paratuberculosis using data mining in internal validation by QDA models in 12 water absorbance bands according to the blood plasma reference test and SVM models in total range and 12 water absorbance bands according to two reference tests. In external validation, according to the blood plasma reference test, QDA and SVM models had 100% sensitivity. The best models use milk spectra from QDA or SVM in 12 water absorbance bands with 100% sensitivity in internal and external validation.

By using these aquagrams, the changes in the ratio of water absorbance bands in the milk sample in both healthy and infected groups due to Johne’s disease can be monitored with more accuracy and fast. These changes indicate genetic, physiological and environmental changes due to disease on the water structure in milk. Since all the above conditions are also visible in healthy samples, the comparison of two healthy and infected groups, considering all the conditions governing them, provides the opportunity to select and compare animals. That is the principle of the comprehensive mirror of water.

The accuracy and quality of diagnosis utilizing advanced diagnostic facilities such as aquaphotomics and advanced diagnostic equipment such as near-infrared spectroscopy improves. These facilities assist breeders in detecting Johne’s diseases in the early stages and providing appropriate treatment by simple sample, milk.

## Conclusion

In this study, for the first time, the two groups were chosen based on the results of three consecutive weekly ELISA blood plasma tests and milk ELISA test results as the reference tests in dairy cows by milk samples. The NIR spectrum profile of milk was used to develop three classification models including PCA as an unsupervised method and QDA and SVM as supervised methods, based on the antibody index in plasma or milk and the biochemical changes caused by the disease in the milk, particularly in the structure of the water molecules that make up the milk.

These models were developed in two ranges, in the NIR range 1300–1600 nm and only in 12 water absorbance bands in this range. These models diagnosed Paratuberculosis and distinguished healthy and infected samples with 100% total accuracy, sensitivity, and specificity in internal validation both according to blood plasma and milk ELISA test results as a reference test, especially by SVM models. In external validation, the results of models by 12 water absorbance bands are the same or more accurate than the total range 1300–1600 nm. Additionally, in external validation the results of models according to the blood plasma test results as reference test more accurate than milk ELISA test as reference. These results are the same in QDA and SVM models by 12 water absorbance bands, 20% specificity, 100% sensitivity, and 82% total accuracy. These results could be improved by increasing the number of samples.

Finally, an aquagram was obtained which shows the changes in the 12 water bands in healthy and infected dairy cattle as a multidimensional biomarker. The findings of this study showed that milk NIR spectroscopy and the Aquaphotomics approach can lead to non-invasive, fast, and accurate diagnosis of Paratuberculosis in dairy cattle.

In this study, we found that NIR spectroscopy by supervised methods can be accurate in the diagnosis of Paratuberculosis by milk samples. Completely, we found that Aquaphotomics by using only 12 water absorbance bands of milk can detect the healthy and infected dairy cattle more accurately than the total range. The important result of this study is applied milk sample that easier than blood plasma but classified according to blood plasma ELISA test results with more accuracy than the milk ELISA test. These classifications are completed by using water absorbance bands that decrease the value of data analysis and more accurately, because water is the mirror of biological functions comprehensivly.

In the last, aquagrams showed that Water Absorbance spectral patterns (WASPs) can be used as a multidimensional biomarker to monitor antibody changes in milk, and this method is a very simple, fast, and accurate way to detect healthy and infected dairy cattle by Paratuberculosis. According to the findings of this reasearch and the last findings on the use of blood plasma samples to diagnose Paratuberculosis, the novel methods for the diagnosis of Paratuberculosis in dairy cattle using milk samples with NIR spectroscopy and Aquaphotomics were introduced. The long-term goal of this research team is to create a diagnostic strategy for detection of dairy cattle in the 4 stages and to use more dairy cattle by this non-invasive, accurate, and fast diagnostic method in vivo, which will definitely contribute significantly to the sustainability of genetic resources and global health.

## Materials and methods

This research was approved by the SRBIAU- Institution of Animal Science (SRB − 11-3997) and complied with institutional, national, and international ARRIVE guidelines. All methods were performed in accordance with the relevant guidelines and regulations. The animals studied in this research were not separated from the herd. The farms had a routine program for monitoring JD, and the blood samples taken by staff for ELISA tests were also used for spectroscopy.

The study and analysis of data related to milk samples were presented in two sections. The first section calculates the data based on the blood plasma ELISA testing results as the reference test, and the second section explains the data based on the milk ELISA test results as the reference test.

### Animals and farms

Holstein dairy cows in their second and third lactation were used in this research. It should be noted that the ELISA test was not accrued in the early calf due to the special conditions of Johne’s disease, and the population of that range of cattle is larger than others. Therefore, finding positive cattle is easier. The selected cows were considered free of other diseases, according to the data health file and veterinarian visits. The experiment was carried out over one summer. Two physiologic periods in dairy cattle were chosen, one about 60 days before the drying period from one farm and the other 100–200 days after calving from another farm in Iran’s Isfahan province.

Dairy cows that were to be dried three weeks later were selected randomly from the first farm among the cows in the second and third lactation periods (*n* = 50). In their second or third lactation and about 100 to 200 days after calving, dairy cows were chosen from the second herd (*n* = 50). Dairy cows with similar blood plasma ELISA testing results in three consecutive weeks were chosen based on the history of ELISA testing in the first year. Dairy cows with ELISA test index of less than 20 and greater than 100 were classified as negative and positive Johne’s disease, respectively (Table 2).

**Table 2:** The characteristics of negative and positive animal groups for diagnosis of paratuberculosis according blood plasma and milk ELISA test results as a reference test.

Contents	Herd	Total number of cows	Number of cows according to the blood plasma ELISA test		Number of cows according to the milk ELISA test	
			Positive	Negative	Positive	Negative
60 days before drying	1	8	5	3	6	2
100–200 days after calving (first week)	2	11	8	3	5	6
100–200 days after calving (second week)	2	11	8	3	5	6
100–200 days after calving (third week)	2	11	8	3	5	6
Total	2	19	13	6	11	8

### Blood and milk collection

Four blood samples were collected for each cow in the first farm (*n* = 50*4 = 200), three weeks before the drying period and one week after calving, and from 50 animals, 8 of them were selected according to the similarity in results of ELISA tests. In the second farm, the blood sample tubes were collected from cows whose ELISA tests were confirmed (*n* = 50*3 = 150), and then blood samples were taken three times within three weeks from selected cows according to the similarity ELISA tests (*n* = 11*3 = 33). The blood samples were collected via caudal venipuncture into two commercial blood collection tubes containing the anticoagulant Ethylenediaminetetraacetic acid (EDTA) and immediately placed on ice (*n* = 383). Two tubes were centrifuged at 4000 rpm for 20 min to separate the plasma, and the plasma was stored in six 2 mL microtubes at − 21 °C until NIRS analysis. The second tube was used for the ELISA test with an IDEXX kit. From the selected animals (*n* = 19), in addition to the blood sample, the evening milk samples were collected manually from all 4 glands in equal amounts for three consecutive weeks, after mastitis test and before connecting the milking machine. The samples were quickly divided into two parts. The first part was prepared in the form of 6 microtubes of 2 ml for spectroscopy and kept at −21 °C until the time of spectroscopy. The second part was transferred to the laboratory in three 2 ml microtubes for ELISA testing. All cows had the the same diet (Table 3).

**Table 3:** The number of the dairy cattle selected for diagnosing paratuberculosis using milk samples according to the blood plasma and milk ELISA test results as a reference test.

Contents	The number of animals with positive blood plasma ELISA test	The number of animals with negative blood plasma ELISA test
The number of animals with positive milk ELISA test	9	2
The number of animals with negative milk ELISA test	4	4

In this study, data analysis was conducted within the range of 1300–1600 nm, and subsequently within the 12 water absorbance bands within that range. Table 2 presents the statistical description of the animals investigated for the diagnosis of Johne’s disease using milk samples.

### Reference analysis (ELISA test)

Models were created and evaluated for the selected samples by comparing the estimated and reference values in three results of consecutive blood plasma. The same results of ELISA tests during the three weeks were considered for the reference analysis. In another part of the study, the results of milk ELISA tests of selected animals were considered for the reference analysis.

The IDXX kit, as an ELISA kit, has an enzyme immunoassay for the detection of antibodies directed against MAP in bovine individual serum, plasma, and milk samples. First, obtained coated plates and recorded the sample position. Diluted the Negative Control (NC) 1:20 in the dilution buffer N.12 and dispensed into one well. Diluted the Positive Control (PC) 1:20 in the dilution buffer N.12 and dispensed in two wells. Diluted plasma samples 1:20 in the dilution buffer N.12. After homogenizing the contents, use a microplate shaker. The sample were incubated for 15 min to 2 h at 18–26 °C. Transferred 100µL from each well to the preplate to appropriate wells of the coated microplate. After the contents of the wells were homogenized by a microplate shaker, they were covered and incubated at 18–26 °C for 45 min.

Then the solution was removed and the contents of each well were washed 3–5 times with about 300µL of washing solution. After that,100µL of Conjugate was distributed in each well. Covered it and incubated it for 30 min. Then the solution was removed and each well was washed 3–5 times with approximately 300 µl of solution. Then 100µL of TMB substrate N.9 was placed in each well and incubated for 10 min at 18–26 °C away from direct light. 100µL of stop solution N.3 was dispensed in each well and the optical density values of samples and controls were measured and recorded at 450 nm. The Eq. (1) was used for the calculation of controls and the Eq. (2) was used for plasma and milk samples (IDXX Paratuberculosis screening 06-07130-27 manual):

1 $$\:\mathbf{C}\mathbf{o}\mathbf{n}\mathbf{t}\mathbf{r}\mathbf{o}\mathbf{l}:\:\varvec{P}\varvec{C}\stackrel{-}{\varvec{X}}\:=\frac{\:\varvec{P}\varvec{C}1\left(450\right)+\varvec{P}\varvec{C}2\left(450\right)}{2}$$

$$\:\mathbf{V}\mathbf{a}\mathbf{l}\mathbf{i}\mathbf{d}\mathbf{i}\mathbf{t}\mathbf{y}\:\mathbf{c}\mathbf{r}\mathbf{i}\mathbf{t}\mathbf{e}\mathbf{r}\mathbf{i}\mathbf{a}:\:\frac{\varvec{P}\varvec{C}\stackrel{-}{\varvec{X}}}{\varvec{N}\varvec{C}}\:\varvec{A}\left(450\right)\ge\:3.00\:\text{a}\text{n}\text{d}\:PC\stackrel{-}{X}\ge\:0.350$$

2 $$\:\mathbf{I}\mathbf{n}\mathbf{t}\mathbf{e}\mathbf{r}\mathbf{p}\mathbf{r}\mathbf{e}\mathbf{t}\mathbf{a}\mathbf{t}\mathbf{i}\mathbf{o}\mathbf{n}\:\mathbf{f}\mathbf{o}\mathbf{r}\:\mathbf{p}\mathbf{l}\mathbf{a}\mathbf{s}\mathbf{m}\mathbf{a}\:\mathbf{s}\mathbf{a}\mathbf{m}\mathbf{p}\mathbf{l}\mathbf{e}\mathbf{s}:\:\varvec{S}/\varvec{P}\mathbf{\%}=100\mathbf{*}\frac{\varvec{s}\varvec{a}\varvec{m}\varvec{p}\varvec{l}\varvec{e}\:\varvec{A}\left(450\right)-\varvec{N}\varvec{C}\:\varvec{A}\left(450\right)}{\varvec{P}\varvec{C}\stackrel{-}{\varvec{X}}-\varvec{N}\varvec{C}\:\varvec{A}\left(450\right)}$$

$$\:Negativ:S/P\%<45\%$$

$$\:Suspect:45\%<S/P\%<55\%$$

$$\:Positive:S/P\%\ge\:55\%$$

In this study and to increase the accuracy of the reference test, the animals had the same results of S/P in three consequently test$\:\:\:\ge\:100$, and$\:\:\:\le\:20$ considered positive and negative, respectively. For bovine milk ELISA test,

$$\:Negative:S/P\%<20\%$$

$$\:Suspect:20\%<S/P\%<30\%$$

$$\:Positive:S/P\%\ge\:30\%$$

### NIR spectral signature collection

Milk NIR absorbance spectra were collected using a spectrophotometer (UV-VIS-NIR 3600, Shimadzu CO. Japan) equipped with a quartz cuvette having a 1-mm optical path length (*n* = 111). The samples were thawed over ice for 15 min and warmed between hands for approximately 1 min before NIR spectra collection, and the cuvettes were gently shaken horizontally several times to ensure the uniformity of the milk composition. NIR spectrum acquired in the range of 1280–1630 nm (interval = 0.5 nm; single scan; very slow). Before collecting milk spectra, a reference spectrum was captured from two empty cuvettes, followed by one empty cuvette and one containing distilled water. Three independent spectral signatures were collected per sample, with the cuvette being repacked with milk between each replicate.

### Multivariate analysis (MVA)

The chemometrics-based MVA was performed on the first overtone region of the near-infrared spectrum in the vibrational combination band between 1300 and 1600 nm using Unscrambler X v.10.5. The mathematical pretreatments of the Linear Baseline Correction, Standard Normal Variate (SNV) with detrending polynomial order and a first derivative (symmetric Savitzky–Golay smoothing, points = 12), Smoothing, Normalize, Multiplicative scatter correction (MSC) and Spectroscopic (Absorbance to Transmittance) applied to all the databases described next.

A balanced dataset was created by spectral signatures for each category (healthy or negative, infected or positive) (Table 3). This dataset contains spectra from all 19 dairy cattle and were used to perform principal component analysis (PCA), quadratic discriminant analysis (QDA), Partial least square - discriminant analysis (PLS-DA), and Support Vector Machine (SVM), followed by Aquaphotomics analyses. In the supervised analysis, datasets were created by positive and negative groups to test mathematical preprocessing and modeling bias against the null hypothesis (no biological signature of milk can differentiate between samples from two classes).

The samples were randomly divided into two subsets: a calibration subset for the internal validation set (75%) and a test subset for the external validation set (25%). This calibration equation was derived from various sample sets: each sample set includes a calibration set of positive (> 100) and a calibration set of negative samples (< 20).

### Principal component analysis (PCA)

Principal component analysis (PCA) is an unsupervised multivariate analysis and a well-known statistical method for reducing the dimensionality of data sets, explaining variation in data by ignoring the data label, assisting in pattern detecting in spectral behavior, and finding excluded data^47^. The PCA was applied to the dataset, and the calibration sets were created for the discriminant analysis and completed as the first step to observe spectral features from both negative and positive blood plasma samples to determine dataset groupings and scores distributions, identify dominant peaks in the loadings, and outliers were detected using the Hotelling’s T2 influence plot.

### Discriminant analysis

Discriminant analysis is a supervised and qualitative classification method that can classify new and unknown samples based on separate models for each group. It also helps to interpret differences between groups using linear discriminant analysis (LDA) and nonlinear discriminant analysis like quadratic discriminant analysis (QDA) or partial least squares discriminant analysis (PLS-DA) methods. The LDA is a common technique considering both within-group and between-group variances. Decreasing the dimensionality of data increases the variance between groups and reduces the variance within groups, so that they can be separated from each other. The QDA applies when the variability of each group does not have the same structure (unequal covariance matrix), and the shape of the curve separating groups is not linear^48^. The QDA method was used to describe the nonlinear relationship between groups in raw data and transformed spectra in 1300–1600 nm and 12 water absorbance bands separately. The PLS-DA analysis method was used to recognize the effect layers in discriminant models. All the processes presented above were also implemented for this method. R^2^ and RMSC indices were used to determine the accuracy of the obtained PLS-DA models.

### Support vector machine (SVM) analysis

As a supervised method, support vector machine (SVM) finds an optimal hyperplane or classifier and assigns objects to different classes as correctly as possible. The SVM can effectively avoid over-fitting problems by leaving the most significant possible fraction of points from the same group on the same side and maximize the distance of either group from the hyperplane and structural risk minimum mistake instead of the minimum error of the misclassification on the training set. Due to these advantages, SVM has gained extensive applications, including binary classification^48^.

### Evaluation of classification methods

The quality parameters such as accuracy, sensitivity, and specificity were used to evaluate the performance of classification methods. The sensitivity test quantifies the model’s ability to correctly identify true positive cases of Johne’s disease described by Eq. (3), where TP represents the true positive and FN represents the false negative. A high sensitivity (> 90%) is required when using the prediction model to identify severe but treatable diseases.

The model’s specificity shows its ability to identify healthy samples correctly. The true negative was shown by Eq. (4), where TN represents true negative, and FP represents false positive. Also, the total accuracy is demonstrated by Eq. (5).

3 $$\:Sensitivity\%=(TP/TP\:+\:FN)*100$$

4 $$\:Specificity\%=(TN/TN+\:FP)*100$$

5 $$Total{\text{ }}accuracy\% =\left( {\left( {\left( {TN * \left( {TN/TN\,+\,FP} \right)} \right)+\left( {TP * \left( {TP/TP\,+\,FN} \right)} \right)} \right)/\left( {TN\,+\,FP\,+\,TP\,+\,FN} \right)} \right) * 100$$

### Aquaphotomics

The contribution of water absorbance bands was investigated to detect Positive and Negative blood plasma groups separately in Johne’s disease. Therefore, a two-stage analysis is about all wavelengths of blood plasma samples in the range of 1300–1600 nm (first overtone of water), and the wavelength of only 12 water absorbance bonds in this range were characterized as follows; C1,1336–1348, (2ν3:H2O asymmetric stretching vibration), C2,1360–1366,(OH–·(H2O)1,2,4: Water solvation shell), C3, 1370–1376 (ν1 + ν3: H_2_O symmetrical stretching vibration and H_2_O, asymmetric stretching vibration ), C4, 1380–1388(OH–·(H_2_O)1,4: Water solvation shell, O_2−_·(H_2_O)4: Hydrated superoxide clusters, 2ν1: H_2_O symmetrical stretching vibration ), C5, 1398–1418,( Water confined in a local field of ions (trapped water), S0: Free water, Water with free OH–), C6,1421–1430 (Water hydration band, H–OH bend and O–HO), C7, 1432–1444, (S1: Water molecules with 1 hydrogen bond), C8, 1448–1454, (OH–·(H_2_O)4,5: Water solvation shell ), C9, 1458–1468, (S2: Water molecules with 2 hydrogen bonds, 2ν2 + ν3: H_2_O bending and asymmetrical stretching vibration), C10, 1472–1482, (S3: Water molecules with 3 hydrogen bonds), C11, 1482–1495, (S4: Water molecules with 4 hydrogen bonds), C12, 1506–1516,(ν1: H_2_O symmetrical stretching vibration, ν2: H_2_O bending vibration, strongly bound water)3. Finally, the water bond study results were shown by aquagram using Eq. (6)^25^.

6 $$\:{A}^{{\prime\:}}\lambda\:=(A\lambda\:-\:\mu\:\lambda\:)/\sigma\:\lambda\:\:\:\:\:$$

Where A’ λ is the normalized absorbance value displayed on the radar axis; Aλ is absorbance after scatter correction (multiplicative scatter correction using the mean of the dataset as a reference spectrum or standard normal variant transformation); µλ is the mean of all spectral; σλ is the standard deviation of all spectral; and λ are the selected wavelengths from WAMACS regions corresponding to the activated water absorbance bands^25^.

## Data Availability

The datasets generated or analyzed during the current study are available from Abbas Pakdel or Reza Massudi on reasonable request (pakdel@iut.ac.ir, r-massudi@sbu.ac.ir).
